# Selecting informative genes for discriminant analysis using multigene expression profiles

**DOI:** 10.1186/1471-2164-9-S2-S14

**Published:** 2008-09-16

**Authors:** Xin Yan, Tian Zheng

**Affiliations:** 1 Russell Investments, Tacoma, WA, USA; 2 Department of Statistics, Columbia University, New York, NY, USA

## Abstract

**Background:**

Gene expression data extracted from microarray experiments have been used to study the difference between mRNA abundance of genes under different conditions. In one of such experiments, thousands of genes are measured simultaneously, which provides a high-dimensional feature space for discriminating between different sample classes. However, most of these dimensions are not informative about the between-class difference, and add noises to the discriminant analysis.

**Results:**

In this paper we propose and study feature selection methods that evaluate the "informativeness" of a set of genes. Two measures of information based on multigene expression profiles are considered for a backward information-driven screening approach for selecting important gene features. By considering multigene expression profiles, we are able to utilize interaction information among these genes. Using a breast cancer data, we illustrate our methods and compare them to the performance of existing methods.

**Conclusion:**

We illustrate in this paper that methods considering gene-gene interactions have better classification power in gene expression analysis. In our results, we identify important genes with relative large p-values from single gene tests. This indicates that these are genes with weak marginal information but strong interaction information, which will be overlooked by strategies that only examine individual genes.

## Introduction

Gene expression data that measure mRNA abundance in samples under different conditions provide a valuable tool for studying the difference between the molecular activities of an organism under these conditions [1,2]. Such a study is usually based on a discriminant analysis of the sample classes (under different "conditions") using the gene expression profiles observed in the experiments. Because of the large number of genes that are measured in one microarray experiment, a critical step is to select the genes that are informative about the between-class difference. Such a selection also allows researchers to identify genes that are potentially relevant to the between-class difference in the molecular activities.

The most popular strategy of selecting informative genes is to use *t*-type scores that compute the mean expression difference of a gene between two classes, standardized by a measure of within-class variability [1,3,4]. Similar strategies have also been used, motivated by the *t*-type scores' relevance to the two-sample mean test, such as the Wilcoxon test and the maximum invariant test, etc. [5-7]. In van't Veer et al. (2002) [2], the correlation coefficient computed between a gene's expression and the class label (0 and 1) was directly used. This can also be shown as a *t*-type score using a different standardizing measure of variability. The common advantage of these tests are their simplicity. The disadvantage is that they only evaluate the genes individually (or marginally) and ignore possible class information contained in gene-gene interactions. Dudoit et al. (2002) [8] carried out a comparison of current discriminant analysis methods and commented on the lack of gene selection methods that consider interactions among genes.

In this paper, we propose and study a framework of selecting informative genes via backward information-driven search on gene sets. The central idea of this framework relies on between-class information measures defined on multi-gene expression profiles. We first consider the *Multigene Profile Association *(MPAS) method [9] that was adapted from the Backward Genotype-Trait Association (BGTA) method derived in [10] for gene mapping. For the analysis of gene expression, the innovation lies in the discretization of the original expression values of genes into *discrete expression state *values and the definition of *multigene expression state *profiles. The discretization is done through k-means clustering on the training set, where the expression values of a gene are clustered into three levels: *high, normal*, or *low*. Such a discretization greatly reduces the complexity of the data and make the formulation of multigene profiles feasible. It also makes the analysis more resistant to outliers and extreme values. Once the discretization is applied to the gene expression levels, MPAS information measure is readily defined (similar as in [10]) that measures the association between a partition of samples by the multigene expression state profiles and the class label.

Although MPAS has demonstrated improved discriminant power in our evaluation, its performance depends on the number of states into which the expression values are discretized. In our experiment with MPAS, we have used an intuitive choice of three levels (*high*, *normal *or *low*). However, the performance could be improved by using a more refined definition of states. It could also be possible that different genes or data sets may require different "optimal" numbers of states. To avoid such arbitrary choice, we consider a between-class difference information measure directly defined on the original expression values.

The second information-driven method we propose is the *signed *Multigene Profile Association (sMPAS) method. The derivation of sMPAS comes from the methods for marked point processes (MPP) [11]. Considering the space of multi-gene expression profiles spanned by several genes, the discriminant analysis between two classes (cancer versus normal, for example) is equivalent to the spatial segregation problem for two point processes with different labels. In spatial statistics, the nearest neighbor distance (NND) has been used as a good indicator of separation between clusters of points (e.g., [12,13]). Therefore, for each point in the training set with *n *observations, we compute its distance to the nearest neighbors in the two sample classes respectively. This gives us *n *pairs of distances, the distance to the nearest neighbor of the same class and the distance to the nearest neighbor of the other class. The sMPAS information score is than defined as the *sign test *[14,15] statistic defined on these *n *pairs of distances. For genes whose expression values segregate one sample class from the other, sMPAS information score is greater than expected by chance, reflecting the importance of these genes for the discriminant analysis task.

On a training set, our approach examines the large set of genes in a microarray study through repeated backward elimination screenings on small random sets of genes one at a time. For each random subset, the information measures are evaluated based on the expression profiles of these genes. Genes are removed recursively from the current set to increase the information measures until no improvement can be achieved. The retained genes from each screening are recorded. After the process is repeated a large number of times on different random sets of genes, the genes are then ranked based on their aggregated return frequencies. It should be noted that our backward recursive elimination is different from Li and Yang (2005) [16]. Li and Yang (2005) [16] proposed to eliminate redundant genes by using inter-gene association whereas our elimination is based on gene's contribution to an information score.

For class prediction based on the *informative genes *selected, a *neighborhood voting *method is naturally formulated. For an inquiry sample, a *vote *is given by a particular informative gene, according to the class dominance of the training data in the *neighborhood *of this inquiry sample. The neighborhood of this inquiry sample is defined to be its expression state on this given gene for MPAS and its nearest neighbor for sMPAS. The corresponding *weight *for this *vote *reflects the differentiating power of this given gene with respect to the two classes. *Votes *are calculated for all selected top genes and aggregated using corresponding *weights*.

We evaluate and compare the performance of MPAS and sMPAS with several conventional methods including t statistic [3], gene voting [1], SAM (Significance Analysis of Microarrays) [4] and correlation score [2], using the training set studied by van't Veer et al. (2002) [2] that consists of 78 breast cancer patients. Under a 13-fold cross-validation framework, our information-driven approach demonstrates advantages and better performance over these methods (~20% improvement) through the efficient usage of interaction information. Interestingly, different from other methods evaluated, MPAS and sMPAS select and use a substantial number of genes with large marginal p-values, which would be overlooked by individual-gene methods.

## Methods

To facilitate discussion, suppose that we have a set of training data that consists of *n *samples, *n*_1 _of which are from class I and *n*_2 _= *n *- *n*_1 _of which are from class II. In the microarray experiments, a total of *P *genes are measured.

### Multigene profile association (MPAS) method

**Figure 1:**
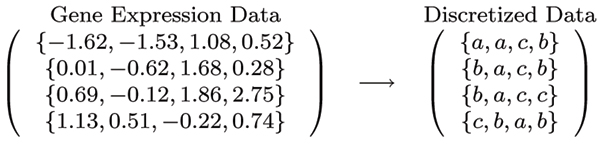
Use k-means to discretize expression values.

As discussed in the *Introduction *section, we discretize each gene into three states "a","b" and "c", representing *low *(under-expressed), *normal*, and *high *(over-expressed) respectively. More specifically, we apply k-means clustering to a gene's expression values in the training set with classes I and II pooled, setting the initial centers to be the minimum, the median and the maximum as illustrated in Figure 1. After the discretization, the data consist of genes that each with three states and we are to identify the important genes that capture the difference between class I and class II.

In Zheng et al. (2006) [10], backward genotype-trait association (BGTA), a backward screening algorithm was developed for gene mapping in case-control studies on complex diseases. In such studies, association is evaluated between a dichotomous disease trait with genome loci, each with three genotypes. BGTA considers interactions between loci when evaluating the association and therefore has better power in detecting important genes for complex diseases. In the following, we directly apply the BGTA framework on the discretized gene expression values and derive the gene selection process and corresponding classifier.

Consider *K *genes, a given sample corresponds to a *K*-tuple vector with expression states as its elements, which is defined as a K-gene *multigene profile *on these genes. Thus for a fixed value of *K*, there are a total of *T *= 3^*K *^patterns possible. We measure the association between this set of *K *genes with the class label using a *multigene profile difference *defined as,

$$\text{MPD}=\sum_{i=1}^{T}{\left(w_{1}*n_{i1}-w_{2}*n_{i2}\right)}^{2}$$

where *w*1 = *n*_2/(*n*_1 + *n*+ 2), *w*_2 _= *n*_1_/(*n*_1 _+ *n*_2_), *n*_*i*1 _is the number of profile *P*_*i *_observed among class I samples and *n*_*i*2 _is similarly defined for class II. This is a straightforward adaptation of the genotype-trait distortion (GTD) score used in BGTA. To evaluate the informativeness of a given gene, say *G*_*i*_, among the *K *genes, we recalculate a MPD score on expression profiles with this gene removed. The main statistic in the MPAS method, *Multigene Profile Association Score *for a given gene among *K *genes, is then defined as,

(1) $$\text{MPAS}(G_{i}|\text{current genes})=\frac{1}{2}\Delta \text{MPD}(G_{i}\text{removed})+\delta$$

where ΔMPD(*G*_*i*_removed) = MPD(*G*_*i*_removed) - MPD and *δ *is an adjusting term so that MPAS has an expectation of 0 under the null hypothesis that this gene has no association with the class difference.

This is a straightforward adaptation of the genotype-trait association (GTA) score used in BGTA. More computational details can be found in [10]. Similar to GTA, MPAS measures the importance of each gene in terms of its association with the class label, given current genes. Positive value of MPAS suggests that the deletion of *G*_*i *_reduces noise and boosts information measured by MPD and negative value means information loss.

Based the MPAS, we designed a backward eliminating process for selecting important genes, similar to BGTA in [10]. The gene selection process using MPAS:

1. Run *B *iterations on random subsets of genes.

(a) For the *b*^*th *^iteration, randomly pick a subset of *K *genes, *S*_*b *_out of the *P *genes in a given study to construct the initial *K*-gene profiles. *P *is usually in the thousands.

(b) For each gene in *S*_*b*_, compute MPAS_*r*_.

(c) If all genes in *S*_*b *_have negative MPAS_*r *_scores, stop current iteration. Otherwise, remove from *S*_*b *_the gene with the highest positive MPAS and iterate back to step 1b.

(d) Record retained genes in the final set of *S*_*b*_.

2. After *B *iterations, compute the cumulative selection frequency for each gene, *F *= (*F*_1_,*F*_2_,..., *F*_*P*_).

3. Select *p *genes with the highest selection frequencies.

The random subset selection procedure takes advantage of the aggregated importance of a gene measured by the MPAS score. This strategy was evaluated in [17] and [10] using simulated data, where it was shown that genes with higher importance have higher overall chance to be retained by such a screening algorithm. In the validation example, we used *B *= 500000 and *K *= 10.

### MPAS class predictor

For class prediction, we propose to use a classifier similar to that used in [1], as a *weighted sum of votes*, with "weights" being a gene's (or gene pair's) level of importance and "vote" being the gene's (or gene pair's) prediction on a particular inquiry sample. In this study, the weighted sum uses both individual genes (marginal predictors) and gene pairs (joint predictors). Detailed construction of a MPAS predictor is outlined as follows.

Once *p *top genes were determined based on their selection frequencies. They are to be used as marginal predictors first. Their marginal weights are defined as $W_{i}^{\left(m\right)}=F_{i}/\sum_{i=1}^{p}F_{i}$. Take an inquire sample with expression values **x **= {*x*_1_,..., *x*_*p*_} on these selected *p *genes, the expression values, *x*_*i*_'s, are first discretized using the k-means result on the training data. Say, for gene *i*, inquiry sample **x **falls in state *h *(*h *takes values *a*, *b *or *c*). The vote of gene *i *towards **x **is then *V*_*i *_^(*m*) ^= *w*_1 _*Q_*i *_^*h*,1^/Q_*i *_^*h *^where *Q*_*i *_^*h *^= *w*_1 _*Q_*i *_^*h*,1 ^+ *w*_2 _*Q_*i *_^*h*,2^, is the adjusted total number of training samples with gene *i*'s state being *h*, with *Q*_*i *_^*h*,1 ^and *Q*_*i *_^*h*,2 ^being the numbers of class I and class II samples with gene *i*'s state being *h*, respectively; *w*_1 _and *w*_2 _are the sample size weights as defined previously in (). The marginal vote for **x **is then

(2) $$P^{\left(m\right)}(x\text{ belongs to class I}|y)=\sum_{i=1}^{p}W_{i}^{\left(m\right)}V_{i}^{\left(m\right)}.$$

The class prediction is class I if *P*^(*m*)^(*x *belongs to class I|*y*) ≥ 0.5, and class II otherwise.

To train joint predictors, the MPAS screening process is run a second time on the *p *selected genes only. Cumulative selection frequencies are collected for each pair of genes: ${\tilde{F}}_{i},i=1,2,...,\left(\begin{array}{c} p\\ 2 \end{array}\right)$. Among these pairs, rated by their selection frequencies, top p* pairs are to be used as joint predictors. For each selected pair, the weight $W_{i}^{\left(j\right)}={\tilde{F}}_{i}/\sum_{i=1}^{p^{*}}{\tilde{F}}_{i}$ and the joint vote $V_{i}^{\left(j\right)}=w_{1}*{\tilde{Q}}_{i}^{h,1}/{\tilde{Q}}_{i}^{h}$ are similarly defined as for the marginal predictors, except that the state takes pairs of values, i.e., *h *∈ {(*a*, *a*), (*a*, *b*), (*a*, *c*), (*b*, *a*), (*b*, *b*), (*b*, *c*), (*c*, *a*), (*c*, *b*), (*c*, *c*)}. The joint vote is then the weighted sum of votes from these joint predictors,

(3) $$P^{\left(j\right)}(x\text{ belongs to class I}|y)=\sum_{i=1}^{p^{*}}W_{i}^{\left(j\right)}V_{i}^{\left(i\right)}.$$

Finally, the marginal and joint votes are combined into the MPAS predictor as follows:

(4) $$\begin{array}{l} P(x\text{ belongs to class I}|y)\\ =\alpha P^{\left(m\right)}(x\text{ belongs to class I}|y)\\ +(1-\alpha )P^{\left(j\right)}(x\text{ belongs to class I}|y) \end{array}$$

where 0 ≤ *α *≤ 1 is a constant we use to weigh the contribution from the marginal vote and the joint vote. In the validation section, we have used 50 for both *p *and p*, with *α *= 0.75 for validation. Here we have chosen the values of *p *and p* to make the number of features selected comparable to the other methods (e.g., [1]). *α *= 0.75 was chosen to put more weights on the marginal vote, which tends to be less overfitting than the joint vote. In future practice, when the size of the data allows, we plan to use cross-validation within the training set to select *p*, p* and *α*.

### Signed Multigene Profile Association (sMPAS) method

In the previous section, we proposed the use of the multigene expression state profiles for studying association between a set of genes and the class label. Here, the expression state is obtained through discretization by k-means clustering. The number of states needs to be specified for the k-means algorithm. Without any prior knowledge on what is an appropriate number of states, the choice is relative arbitrary. It is also possible that the number of *natural *expression states is different for different genes. In a data-rich situation, a good estimation of the gene expression's density function can shed light on this issue. However, the sample size is usually very small for gene expression studies. It is then desirable to avoid such an arbitrary choice in evaluating the importance of genes. Moreover, converting the original continuous expression data into discrete values might result in loss of information. In this section, we propose the signed Multigene Profile Association (sMPAS) method, which incorporates the continuous gene expression values into a multigene expression profile score.

We start the derivation of sMPAS by first restating the feature selection problem using the spatial segregation notations. Each individual sample in the training set can be treated as a spatial point, each with a class label (eg. cancer or normal), while the expression values of the genes under study decide the coordinates of each point. In other words, each gene under study corresponds to one dimension of the space of multi-gene expression profiles, where the two classes of points (samples) are to be segregated. Thus, the original gene selection problem can be studied as the dimension selection problem for an optimal spatial segregation. Thus, searching for the informative genes that are associated with the class differentiation, is equivalent to searching for the subset of dimensions for an "ideal pattern", under which the neighborhood (defined in the multi-gene expression profile space) of a class I individual contains mostly (if not exclusively) points with class I labels.

Considering *K *genes under study, the *j*^*th *^sample from class I has expression profile

$$X_{j}^{\left(I\right)}={\left(x_{1j}^{\left(I\right)},x_{2j}^{\left(I\right)},...,x_{Kj}^{\left(I\right)}\right)}^{t}.$$

Similarly, we denote a sample from class II as *X*_*l *_^(*II*)^. The two marked point processes to be segregated are denoted as *X*^(*I*) ^and *X*^(*II*)^, corresponding to the two classes respectively. If these *K *genes segregate class I points from class II points, we would expect the proportion of *X*^(*I*) ^points in the neighborhood of any fixed *X*^(*I*) ^point is greater than that expected by chance.

Given a fixed point *X*_*j*_^(*I*) ^∈ *X*^(*I*)^, define

$$\begin{array}{cc} \nu (X_{j}^{\left(I\right)}=\underset{X_{s}^{\left(I\right)}\in X^{\left(I\right)}}{\inf}||X_{j}^{\left(I\right)}-X_{s}^{\left(I\right)}|{|}_{K} & s\neq j \end{array}$$

as its Euclidean distance to the nearest neighbor among the other points in the same class as itself (that is, class I). And define

(5) $$\tau (X_{j}^{\left(I\right)}=\underset{X_{l}^{\left(II\right)}\in X^{\left(II\right)}}{\inf}||X_{j}^{\left(I\right)}-X_{l}^{\left(II\right)}|{|}_{K}$$

as its distance to the nearest point that belongs to the other class (*X*^(*II*)^). Here the distance is computed in the *K*-dimensional space spanned by the *K *genes under study.

Under the null hypothesis that *X*^(*I*) ^and *X*^(*II*) ^are not spatially segregated, points from class I and class II can be approximately regarded as two independent point processes with their intensity ratio being (*n*_1 _- 1)/*n*_2 _at *X*_*j*_^(*I*)^. As a result, the probability that *ν*(*X*_*j*_^(*I*)^) ≥ *τ*(*X*_*j *_^(*I*)^) is (*n*_1 _- 1)/(*n *- 1). If the density of class I points is higher than class II at *X*_*j *_^(*I*) ^(that is, the class I points are distributed tightly away from class II, in the feature space spanned by current genes under study), the probability of having a class I nearest neighbor is greater than (*n*_1 _- 1)/(*n *- 1). We therefore define the signed Multigene Profile Information (sMPI) score as a sign test statistic [14,15] defined on (*ν*(*X*_*j*_^(*I*)^), *τ*(*X*_*j *_^(*I*)^)), that is

$$\text{sMPI}(X^{\left(I\right)})=\sum_{j=1}^{n_{1}}1_{\left\{\nu (X_{j}^{\left(I\right)})\geq \tau (X_{j}^{\left(I\right)})\right\}},$$

where **1**_{·} _is the indicator function. sMPI is a non-negative integer between 0 and *n*_1_. This is also equivalent to counting the correct predictions of the nearest neighbor classifier for class I using leave-one-out validation.

For the information-driven screening, we define sMPAS for gene *G*_*i *_in current evaluation set as the difference between the sMPI scores without and with *G*_*i*_, that is,

$$\begin{array}{ll} & \text{sMPAS}(G_{i},X^{\left(I\right)}|\text{current genes})\\ = & \Delta \text{sMPI}(X^{\left(I\right)})(G_{i}\text{removed}). \end{array}$$

The above sMPAS is an integer between -*n*_1 _and *n*_1_, with a negative value indicating the importance of *G*_*i *_in terms of informativeness about *X*^(*I*) ^against *X*^(*II*)^. Using results on point processes and some simplifying assumptions, we can prove that sMPAS has non-negative expectation if *G*_*i *_is not informative about the between-class difference (not shown here).

The information measure and association score can be similarly defined with points in *X*^(*II*) ^as well. These statistics are then to evaluate the informativeness of genes about class II against class I.

A similar backward elimination screening algorithm as that used in MPAS is then applied using the scores defined above. The only difference is that we run the screening twice, first using the scores for *X*^(*I*) ^and then using the scores for *X*^(*II*)^. During the screening, sMPAS captures dimensions where the two classes are segregated, and the segregation of points is very sensitive to the dimensions considered. It is therefore more important to track the specific interacting dimensions during the screening. Instead of counting important genes, for sMPAS, we calculated the number of times a pair of genes were retained and select top *p *pairs of genes for the predictor, half of which for class I and half for class II. For the validation example, we choose top 50 pairs of genes according to the sMPAS method.

### sMPAS class predictor

For an inquiry sample *x*, each selected pair of informative genes generates a signed vote depending on *x*'s nearest neighbor from the training set. In the space spanned by the *i*^*th *^pair of informative genes, identify the nearest neighbor to *x *from the training data, say with distance NND_*i*_(*x*) away from *x*. The vote from this pair of genes is then

$$V_{i}(x)=\text{sign}(\text{NN})\frac{1}{1+{\text{NND}}_{i}(x)},$$

where sign(NN) is 1 if the nearest neighbor in the training set is from class I and is -1 if the nearest neighbor is from class II.

The weight of this pair of genes' vote should depend on their information score sMPI_*i*_. For pairs selected for class I points, sMPI score is between 0 and *n*_1_. We propose to use weight

$$W_{i}=\sum_{k=1}^{{\text{sMPI}}_{i}}\left(\begin{array}{c} n_{1}\\ k \end{array}\right)\theta_{1}^{k}{\left(1-\theta_{1}\right)}^{n_{1}-k},$$

where *θ*_1 _= (*n*_1 _- 1)/(*n *- 1). Assume a random variable X follows the Binomial distribution with size *n*_1 _and probability *θ*_1_. The weight *W*_*i *_is the probability that *X *is less than or equal to the sMPI_*i *_observed. This is one minus the p-value of sMPI_*i *_as a sign test statistic. For pairs of informative genes selected for class II, we define the weight similarly,

$$W_{i}=\sum_{k=1}^{{\text{sMPI}}_{i}}\left(\begin{array}{c} n_{2}\\ k \end{array}\right)\theta_{2}^{k}{\left(1-\theta_{2}\right)}^{n_{2}-k},$$

where *θ*_2 _= (*n*_2 _- 1)/(*n *- 1).

Given the votes and their weights, we classify the inquiry sample *x *to class I if and only if:

$$\sum_{i=1}^{p}W_{i}V_{i}(x)\geq 0.$$

## Results

### The breast cancer data

It does not seem appropriate to exam our approach using simulation method due to the lack of commonly recognized statistical models for large-dimensional gene expression data. For the purpose of validation, we applied MPAS and sMPAS, as well as other measures to the breast cancer data studied by van't Veer et al. (2002) [2]. We choose single gene strategies: p-value from the two-sample *t *test, the Gene Voting method [1], SAM [4] and the correlation coefficient [2] for comparison to illustrate the information contained in multigene expression profiles.

In van't Veer et al. (2002) [2], expression values of 24,881 genes were measured for 44 good prognosis breast cancer samples (class I samples) and 34 poor prognosis breast cancer samples (class II samples). In the original paper, the authors selected 4936 genes after preliminary analysis. Here, according to [2], preliminary analysis includes the elimination and imputation for missing values in original experiment data. Redundant genes were eliminated using rules of fold changes and P-values as well. However, since they were not allowed to disclose the result on these 4936 genes, we used the 4918 genes obtained by Tibshirani et al. (2002) [18]. Please refer to reference [18] for detailed information.

For convenience, each gene was standardized by its mean and standard deviation, so that a gene would have mean 0 and variance 1 across individuals. To create a fair comparison, equal sizes of top ranked genes or gene pairs (50 in our analysis) by each gene selection measure were taken to construct classifiers. For a measure without a specified classifier, such as t Statistics and SAM, DLDA was used to make predictions.

### Validation results

**Figure 2:**
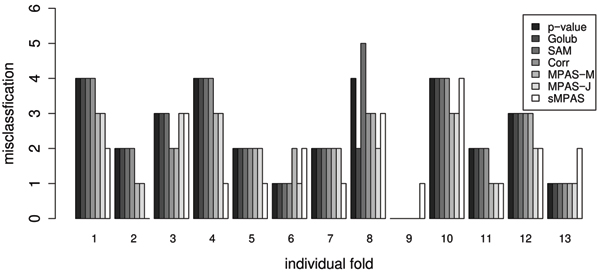
Misclassification for each of the 13 folds using all 7 predictors.

To avoid the under-estimation of prediction error using only the training set, we followed the guideline in [18] and carried out a 13-fold cross-validation on the data to evaluate the gene selection methods and their corresponding predictors as follows: 1) Divide the 78 cases into *K *= 13 equal-sized folds of 6 cases each; 2) Set aside one of the folds. Using only the data from the other 12 folds to select the top 50 genes or gene pairs ranked by each gene selection measure; 3) Use the corresponding predictor of each gene selection method to predict the class labels for the 13th fold; 4) Calculate a total misclassification number for each of the predictors. Summary for the prediction error rate over all 78 cases was given in Table 1. Prediction errors for each of the 13 folds were plotted in Figure 2.

**Table 1:** Misclassification rates of the evaluated methods on the breast cancer data

Gene Selection	Classifier	Misclassification (Top 50 genes)
P-value of *t*-test	DLDA	0.410
Golub	Golub	0.385
SAM	DLDA	0.423
Corr	Corr	0.385
MPAS	Marginal	0.346
**MPAS**	**MPAS**	**0.308**
**sMPAS**	**sMPAS**	**0.295**

From Table 1, sMPAS has the best overall performance. MPAS based on combined votes is only one misclassification higher than sMPAS. Actually, out of 13 folds, MPAS and sMPAS are tied for performance (tied in three folds; MPAS performs better in five folds and sMPAS performs better in the other five folds). As seen in Figure 2, either MPAS or sMPAS has the best performance in 12 out the 13 folds. The overall performance of sMPAS is a ~20% improvement from the best of the conventional marginal methods. In Table 1 and Figure 2, we also included the performance of the MPAS using only the marginal votes. It is interesting to note that the genes selected by MPAS has better marginal performance than the methods used for comparison.

## Conclusion and discussion

In this paper, we proposed an information-driven gene selection approach for discriminant analysis of gene expression data. The central component of this approach is information measures defined on multiple genes that consider gene-gene interaction. We have compared the empirical prediction performance of genes or gene pairs selected using our approach through a 13-fold cross-validation. A decrease of approximate 20% of misclassification was shown using MPAS and sMPAS, compared with the second-to-best predictors: Golub and correlation score. Prior efforts on this data set are summarized in the Table 2 in Yan and Zheng (2007) [9], which shows that this data set projects a difficult task. The best results came from either one test sample or leave-one-out validation. Evaluation based on one test sample will rely on the specific data splitting heavily. Leave-one-out tends to under-estimate the misclassification rate. One example can be found in [19]: the LOO-CV kPCA using radial basis function had perfect performance on leave-one-out validation but had 0.632 misclassification rate on an independent test sample. Considering these factors, the classification performance using genes selected by MPAS is excellent, even compared with methods such as the support vector machine (SVM).

**Figure 3:**
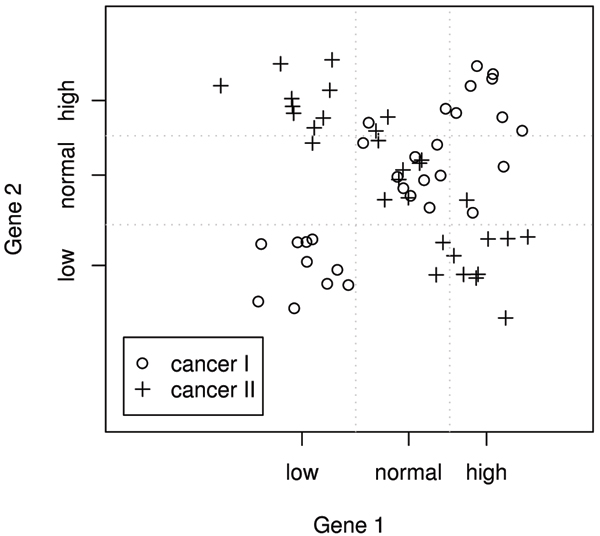
**A hypothetical example of gene interactions**. Cancer I individuals have a clear pattern of being (Low, Low) or (High, High) while Cancer II individuals share the opposite trend. These two genes would not be regarded as important, if evaluated only marginally.

In addition to the improved classification performance, the more interesting feature of MPAS and sMPAS is the consideration of higher-order gene interactions. By converting original data into discrete states, MPAS potentially loses some information. However, such reduction of complexity allows us to look at multigene profiles by adopting an existing method from genetic epidemiology and extract more interactive information among genes. A simple and straight-forward example can be illustrated in Figure 3, where the assignment of class labels are controlled by two genes jointly, with state distributions (low, low) or (high, high) being nearly exclusively class I and (low, high) or (high, low) being class II. In this extreme case, traditional methods that use marginal signals will not find these two genes important. In the validation example, MPAS demonstrated comparable performance to sMPAS that directly uses the continuous-valued gene expression, which may suggest that the discretization based on k-means clustering managed to retain important information in the microarray data. The sMPAS method has the advantage of its direct use of gene expression values without loss of information. The current form of the method uses the Euclidean distance, which makes it less robust to extreme values and outliers. This may explain its less stable classification performance shown in Figure 2. Due to its motivation from the theory on marked point processes, our theoretical inference (not shown in this paper) suggests sMPAS may turn out to be a much more powerful method for data of larger sample sizes.

**Figure 4:**
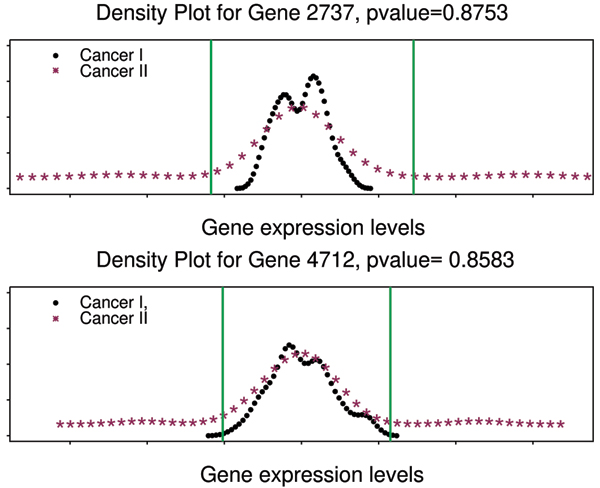
**Density curves for large p-valued genes**. Vertical lines indicate the cut-offs produced by k-means. Gene 2737 (LOC51002) is annotated as a "CGI-121 protein". Gene 4712 (DAXX) is annotated as "death-associated with protein 6".

**Figure 5:**
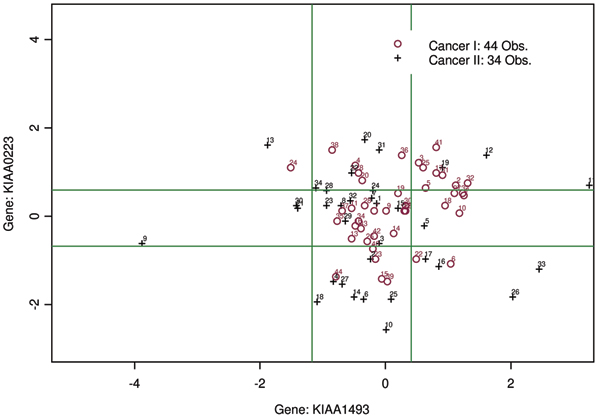
**Joint pattern of a gene pair identified by sMPAS: gene KIAA1493 and gene KIAA0223**. Green solid lines indicate the k-means partition of these two genes. KIAA1493 and KIAA0223 have marginal p-values 0.16 and 0.07 respectively, and jointly their sMPI score has p-value 0.0013.

Among the genes selected by MPAS and sMPAS, some have unusually large p-values. We further investigated this matter by plotting some of these gene's density curves. (Figure 4). From these plots, we can see that, with cancer II samples having much fatter tails in both directions, there are indeed distinguishable patterns between the two cancer classes. However, with almost identical means, such information would be ignored by marginal predictors such as the *t*-test. Results from MPAS and sMPAS also indicate that some genes are jointly returned more than randomly. For instance, gene 4226 (RPS6) were retained together with gene 844 (EST: Contig24094RC) almost 2.4 times more than expected by chance. In Figure 5, we plotted the joint distribution of gene expression values of KIAA1493 and KIAA0223. This pair of genes is regarded as informative about cancer I by the sMPAS method. It is shown that cancer I points distribute tightly in the space spanned by these two genes. Such association in the results of MPAS and sMPAS may lead to interesting hypothesis for further biological studies on these genes.

Solid lines in Figure 5 indicate the k-means cut-off thresholds for MPAS, which suggest the expression state profiles of these two genes used by MPAS are not very informative about the class difference. This clearly demonstrates the advantage of sMPAS. On the other hand, MPAS demonstrated more stable performance than sMPAS in Figure 2. Some combination of these two strategies should be considered in the future to further improve the performance and to identify more gene-gene interactions.

## Competing interests

The authors declare that they have no competing interests.

## Authors' contributions

XY and TZ designed the research, carried out the research and data analysis, and wrote the paper. All authors read and approved the final manuscript.

## References

[B1] Golub TR, Slonim DK, Tamayo P, Huard C, Gaasenbeek M, Mesirov JP, Coller H, Loh ML, Downing JR, Caligiuri MA, Bloomfield CD, Lander ES (1999). Molecular classification of cancer: Class discovery and class prediction by gene expression monitoring. Science.

[B2] van't Veer LJ, Dai HY, Vijver MJ van de, He YDD, Hart AAM, Mao M, Peterse HL, Kooy K van der, Marton MJ, Witteveen AT, Schreiber GJ, Kerkhoven RM, Roberts C, Linsley PS, Bernards R, Friend SH (2002). Gene expression profiling predicts clinical outcome of breast cancer. Nature.

[B3] Cui XQ, Churchill GA (2003). Statistical tests for differential expression in cDNA microarray experiments. Genome Biology.

[B4] Tusher VG, Tibshirani R, Chu G (2001). Significance analysis of microarrays applied to the ionizing radiation response. Proc Natl Acad Sci USA.

[B5] Efron B, Tibshirani R (2002). Empirical Bayes methods and false discovery rates for microarrays. Genetic Epidemiology.

[B6] Efron B, Tibshirani R, Storey JD, Tusher V (2001). Empirical Bayes analysis of a microarray experiment. Journal of the American Statistical Association.

[B7] Schadt EE, Li C, Ellis B, Wong WH (2001). Feature extraction and normalization algorithms for high-density oligonucleotide gene expression array data. Journal of Cellular Biochemistry.

[B8] Dudoit S, Fridlyand J, Speed TP (2002). Comparison of discrimination methods for the classification of tumors using gene expression data. Journal of the American Statistical Association.

[B9] Yan X, Zheng T (2007). Discriminant analysis using multigene expression profiles in classification of breast cancer. Proceedings of the 2007 International Conference on Bioinformatics and Computational Biology (BIO-COMP'07).

[B10] Zheng T, Wang H, Lo S (2006). Backward Genotype-Trait Association (BGTA)-Based Dissection of Complex Traits in Case-Control Designs. Human Heredity.

[B11] Stoyan D, Kendall WS, Mecke J (1995). Stochastic Geometry and its Applications.

[B12] Dixon P (1948). Testing spatial segregation using a nearest-neighbor contingency table. Ecology.

[B13] Ripley BD (1979). Tests of 'randomness' for spatial point patterns. Journal of the Royal Statistics Society: Series B.

[B14] Dixon WJ, Mood AM The Statistical Sign Test. Journal of the American Statistical Association.

[B15] Hodges JLJ (1955). A bivariate sign test. The Annals of Mathematical Statistics.

[B16] Li F, Yang YM (2005). Analysis of recursive gene selection approaches from microarray data. Bioinformatics.

[B17] Lo SH, Zheng T (2002). Backward Haplotype Transmission Association (BHTA) algorithm – a fast multiple-marker screening method. Human Heredity.

[B18] Tibshirani R, Efron B (2002). Pre-validation and inference in microarrays. Statistical Applications in Genetics and Molecular Biology.

[B19] Pochet N, De Smet F, Suykens JAK, De Moor BLR (2004). Systematic benchmarking of microarray data classification: assessing the role of non-linearity and dimensionality reduction. Bioinformatics.

